# Coexistence of medullary and papillary thyroid carcinoma in the same lobe-isthmus complex: A case report

**DOI:** 10.6026/973206300221959

**Published:** 2026-04-30

**Authors:** Giridhar Murali, Renee Snigdha Arul Anand, Divyanshu Kumar, Chandramouli Marimangalam Premchandrababu, R Sanjay Shankar, Anisha Sivakumar

**Affiliations:** 1 Department of Critical Care, Christian Medical College, Vellore, Tamil Nadu, India; 2 Department of Obstetrics and Gynaecology, Christian Medical College, Vellore, Tamil Nadu, India; 3 Department of General Medicine, Medical College, Kolkata, West Bengal, India; 4 Department of Head and Neck, Madras Medical College and Rajiv Gandhi Government General Hospital, Chennai, Tamil Nadu, India; 5 Department of General Medicine, Madras Medical College, Tamil Nadu, India

**Keywords:** Medullary thyroid carcinoma (MTC), papillary thyroid carcinoma (PTC), mixed thyroid carcinoma, thyroidectomy

## Abstract

The occurrence of coexisting papillary thyroid carcinoma (PTC) and medullary thyroid carcinoma (MTC) in a single thyroid lobe is considered to be uncommon. A 47-year-old male presented with progressive anterior neck swelling. Upon imaging, the diagnosis of papillary thyroid carcinoma was made and the patient underwent total thyroidectomy and right selective neck dissection. Histopathological examination demonstrated that medullary carcinoma was located within the right lobe and follicular variant PTC was located within the left lobe and isthmus regions. Upon evaluation of the cervical lymph nodes, metastatic medullary carcinoma was noted. Confirmation of the presence of calcitonin in the MTC component and thyroglobulin expression in the PTC component through immunohistochemistry illustrated the importance of using meticulous histopathological and immunohistochemical analysis in making accurate diagnoses and providing individualized treatment options.

## Background:

The endocrine system's most prevalent cancer is thyroid carcinoma. The global rise in the rate of thyroid cancer is directly attributable to increases in the capacity to detect and image thyroid cancers [1]. The vast majority of thyroid cancers begin within the follicular epithelium and fall into the differentiated thyroid cancer classification category [2]. The most common subtype of thyroid cancer is papillary thyroid cancer (PTC), which represents 80 to 85% of all thyroid cancers [3]. PTC has a generally indolent disease course and lymphatic metastases to cervical lymphnodes are common; however, the long-term prognosis for patients with PTC is excellent even when they have nodal metastasis [4]. The follicular variant of papillary thyroid carcinoma (FVPTC) is an accepted histologic subtype of PTC. FVPTC has a typical follicular architecture, but some FVPTC variants exhibit infiltrative growth and lymphovascular invasion. Accurate histological identification of these variant thyroid cancers is critical for establishing the optimal treatment plan [5]. Medullary thyroid cancer (MTC) is infrequently diagnosed; it constitutes less than 5% of all thyroid cancers. MTC occurs in parafollicular C cells, which produce calcitonin. The clinical behaviour of MTC is more aggressive than that of differentiated thyroid carcinoma. Lymphatic and hematogenous spread occurs early in the disease and the prognosis is dependent upon the disease stage when diagnosed and the extent of surgical remove [6, 7]. MTC is classified as either sporadic or familial. The germline mutations associated with multiple endocrine neoplasias type 2 are mutations of the RET gene, whereas individuals with sporadic MTC often exhibit somatic mutations of the RET gene. Serum calcitonin is a very sensitive marker for both the diagnosis and monitoring of MTC.

PTC and MTC co-existing synchronously are exceedingly rare, as each of them arises from distinct embryologic origins and molecular pathways. Their synchronous development is referred to as synchronous carcinoma or collision tumor and reported rates of this rarity range from 0.2% to 1% of all thyroid cancers [8, 9]. It is challenging to preoperatively diagnose synchronous pathology; commonly used imaging techniques and cytological preparations identify only the dominant lesion. Cytological preparation of MTC with fine-needle aspirated material may be inadequate for determining whether the patient has MTC. Dual pathology is usually only diagnosed postoperatively at the time of histopathological analysis [10]. The diagnosis of synchronous tumors has major implications with regard to clinical outcomes, as there are significant differences in their postoperative treatment. While radioactive iodine therapy is beneficial for patients with PTC, it is not effective for those with MTC, whereas patients with MTC will benefit from calcitonin monitoring and targeted therapies [11]. Therefore, it is of interest to report a rare case of synchronous medullary and papillary thyroid carcinoma involving the same thyroid gland.

## Case summary:

A 47-year-old male, with a background history of hypothyroidism on hormonal therapy, presented to the Head and Neck Surgery outpatient department with a gradually progressive midline neck swelling over five months. The patient denied symptoms suggestive of hypo- or hyperthyroidism, compressive complaints, or significant family history. He was also a known case of myotonic dystrophy and had undergone pacemaker implantation for atrioventricular block during preoperative evaluation. General examination was unremarkable. On local examination, a solitary, well-defined 3 x 2 cm nodule was palpated in the right thyroid lobe. The nodule extended 1 cm below the thyroid notch superiorly, 2-3 cm above the clavicle inferiorly, reached the anterior border of the sternocleidomastoid laterally and was 1 cm from the midline medially. The overlying skin was normal, with no venous engorgement. The swelling was hard, mobile and non-tender moved on deglutition and did not protrude on tongue protrusion. The trachea was central, with palpable carotid pulsations in normal position. No palpable nodules were present in the left lobe and no cervical lymphadenopathy was appreciated. Neck ultrasound revealed a solitary hypoechoic nodule in the right lobe measuring 26 x 22 x 34 mm, classified as TIRADS IV (Figure 1 - see PDF). A right level II hypoechoic node measuring 11 x 8 x 22 mm, with absent fatty hilum and punctate calcification, was also noted (Figure 2 - see PDF). Fine-needle aspiration cytology (FNAC) from the right lobe was suggestive of follicular variant of papillary thyroid carcinoma (FVPTC). Table 1 (see PDF) provides an overview of the clinical profile and radiological findings of the patient. The patient was a 47-year-old male with significant comorbidities, including hypothyroidism, myotonic dystrophy and atrioventricular block requiring pacemaker support. His primary presentation was a gradually progressive midline neck swelling of five months duration. Fine needle aspiration cytology (FNAC) suggested a follicular variant of papillary thyroid carcinoma (FVPTC). Ultrasonography of the thyroid revealed a suspicious right lobe nodule measuring 26 x 22 x 34 mm, categorized as TIRADS IV. Additionally, ultrasound of the cervical lymph nodes identified a right level II hypoechoic node with loss of fatty hilum and calcifications, consistent with metastatic involvement. The patient underwent total thyroidectomy with right selective neck dissection (levels II-IV) under general anesthesia. Intraoperatively, the right thyroid lobe was completely replaced by a hard nodule, while the left lobe and isthmus appeared atrophic and fibrosed. A firm, rounded, enlarged node was identified at right level IIa. Histopathological examination revealed the right lobe replaced by a 3.6 x 2.4 x 2.3 cm tumor with a tan to haemorrhagic cut surface, abutting the external capsule but without gross extrathyroidal extension. The left lobe and isthmus showed ill-defined fibrotic areas. Microscopy confirmed medullary thyroid carcinoma (MTC) in the right lobe, left lobe and isthmus, with lymphovascular invasion, along with follicular variant of papillary thyroid carcinoma (FVPTC) in the left lobe and isthmus without perineural invasion. Metastatic deposits of MTC were identified in cervical lymph nodes (Figure 3 - see PDF). Histopathological examination demonstrated the simultaneous presence of papillary and medullary thyroid carcinomas within the same thyroid lobes and isthmus, with metastatic involvement of a lymph node by both tumor types. Table 2 (see PDF) summarizes the histopathological and immunohistochemical findings, showing medullary thyroid carcinoma (MTC) in the right lobe and coexistent MTC with follicular variant papillary thyroid carcinoma (FVPTC) in the left lobe and isthmus. Histopathology revealed distinct papillary and medullary thyroid cancers in same thyroid lobes and isthmus, with one lymph node showing metastatic deposits from both tumors. Immunoprofiling revealed calcitonin positivity with absent thyroglobulin expression in MTC areas, while follicular structures stained for thyroglobulin, confirming dual pathology. Immunohistochemistry showed tumor cells positive for calcitonin and negative for thyroglobulin (Figures 4 and 5 - see PDF). The Ki-67 proliferation index was 1-2%.

## Final diagnosis:

MTC, pT2 (m) N1b, maximum tumor size 3.6 cm and FVPTC, pT1a (m) N0, largest focus <1 mm. Postoperatively, PTH and calcium levels were normal, with no hypocalcemic features and intact vocal function. The patient was initiated on thyroid suppression therapy from POD 1 and is currently planned for tyrosine kinase inhibitor (TKI) therapy, calcitonin surveillance and a low-dose RAI scan.

## Discussion:

Papillary thyroid carcinoma (PTC) and medullary thyroid carcinoma (MTC) are both types of thyroid cancer that develop at different stages in an embryo, making them fundamentally different in their embryology. Papillary thyroid carcinoma stems from follicular epithelial cells (found within follicles) that develop from the endoderm. On the other hand, medullary thyroid carcinoma develops from parafollicular C cells, or calcitonin-producing cells, found within the thyroid, which have developed from the neural crest. It is uncommon for PTC and MTC to exist in the same thyroid gland (PTC and MTC tumours coexisting in the same patient) [2, 11]. The coexistence of PTC and MTC in the same patient is referred to as a synchronous collision tumour. Collision tumours exist as two separate neoplasms present within the same gland. The incidence of synchronous or collision tumours is extremely rare. The majority of information we have regarding the presence of both tumours simultaneously comes from either isolated case reports or small patient series [3]. Preoperative diagnosis of synchronous outcomes of both tumours remains a challenge. While fine-needle aspiration cytology (FNAC) is a commonly used method of diagnosing thyroid nodules, FNAC generally identifies only the primary tumour type in the event of a collision tumour [12]. Subsequently, FNAC may miss a second malignancy in the same thyroid gland (as was the case in this patient). FNAC of this patient only identified PTC. Histopathology remains the gold standard for the diagnosis of synchronous tumours. The patient had a diagnosis of FVPTC, located in the left lobe and isthmus of the thyroid and MTC, located within the right lobe of the thyroid, after the patient underwent thyroidectomy. This illustrates the need for a thorough pathological evaluation of the entire thyroidic specimen [13]. Immunohistochemistry plays an integral part in establishing the presence of dual pathologies. For example, calcitonin positivity can be used to confirm the diagnosis of MTC, while thyroglobulin expression can support a diagnosis of PTC. The presence of separate staining patterns indicates two independent tumours and excludes the diagnosis of mixed carcionoma [14]. Histologically, FVPTC can exhibit aggressive, infiltrative behaviour. Awareness of such histological characteristics plays a role in risk stratification and follow-up for this subtype of PTC. MTC, in contrast, tends to exhibit early metastasis to the cervical lymph nodes and has a poor prognosis. The presence of cervical lymph node metastasis in this case reflects the biologically aggressive nature of MTC [15]. The mechanism(s) that lead to the development of synchronous tumours (PTC and MTC) is unclear. Currently, alterations in the RET proto-oncogene have been implicated in both tumour types, with point mutations of the RET gene detected in MTC and RET rearrangements detected in PTC. It is possible that a common genetic susceptibility may contribute to the synchronous development of these tumours [16]. The treatment of patients with synchronous tumours should be developed in a multidisciplinary manner and total thyroidectomy should be performed in patients with both tumours. However, the management strategies for the two tumours are different [17]. Following total thyroidectomy of a patient with PTC, radioactive iodine may be used for follow-up based on iodine uptake in the follicular cells; conversely, MTC does not have radioactive iodide uptake capability [18]. In patients with MTC, calcitonin is used for postoperative monitoring. Hence, advanced therapies such as tyrosine kinase inhibitors may be needed in the future. As such, long-term follow-up for both tumours should occur at the same time to monitor disease recurrence in both tumour types [19]. This case report highlights the limited reports of both PTC and MTC coexisting within the same thyroid gland and underscores the limitations of FNAC as well as the importance of providing comprehensive histopathological and immunohistochemical evaluations of patients with synchronous tumours. By providing early awareness, the follow-up and management of synchronous tumours can be accomplished more efficiently [20]. The primary limitation of this case report is that it is based on a single case report. However, sharing knowledge of rare synchronous tumours improves the awareness of diagnostic options and increases the vigilance of medical professionals regarding the management of patients with synchronous tumours of the thyroid gland.

## Conclusion:

Synchronous medullary and papillary thyroid carcinoma is rare. Accurate diagnosis requires meticulous histopathological and immunohistochemical evaluation. Thus, early recognition enables appropriate multidisciplinary management and surveillance.

## Ethical statement:

The Ethics Committee of Christian Medical College, Vellore, concluded that this case report met the criteria for exemption from Institutional Review Board (IRB) review and there was written informed consent from the patient for publication of this case report and images.

## Availability of data and materials:

The datasets generated and analyzed during the study are available from the corresponding author upon reasonable request.

## Code availability:

Not applicable.Code availability:

## Author's contribution:

Conceptualization: GM, RS.

Investigation: GM, RS.

Methodology: GM, RS.

Project administration: GM.

Resources: GM, RS.

Supervision: GM.

Visualization: GM, RS.

Writing original draft preparation: GM, RS

Writing review & editing: GM, RS.

Approval of final manuscript: GM, RS.

## Funding statement:

No funding to declare

## References

[1] Yang DS, He J (2025). Front Oncol..

[2] Zhang D (2023). Front Endocrinol (Lausanne)..

[3] Fallahi P (2023). Curr Oncol..

[4] Boucai L (2024). JAMA..

[5] Akgun E (2023). Indian J Cancer..

[6] Hellmann AR (2020). Endokrynol Pol..

[7] Koh HH, Oh YL (2022). J Pathol Transl Med..

[8] Haddad RI (2022). J Natl Compr Canc Netw..

[9] Forma A (2025). Int J Mol Sci..

[10] Hou Y (2024). Nat Commun..

[11] Guda BB (2023). Exp Oncol..

[12] Calapkulu M (2022). J Coll Physicians Surg Pak..

[13] Haddad RI (2025). J Natl Compr Canc Netw..

[14] Samarasinghe S (2020). Endocrinol Diabetes Metab Case Rep..

[15] Milicevic S (2022). Life (Basel)..

[16] Aksoy S (2024). Acta Endocrinol (Buchar)..

[17] Valerio L (2022). Front Endocrinol (Lausanne)..

[18] Mastronikolis N (2020). J BUON..

[19] Lei R (2021). Ultrasound Q..

[20] Hao Z (2024). Medicine (Baltimore)..

